# Disease burden of neonatal invasive Group B *Streptococcus* infection in the Netherlands

**DOI:** 10.1371/journal.pone.0216749

**Published:** 2019-05-09

**Authors:** Brechje de Gier, Merel N. van Kassel, Elisabeth A. M. Sanders, Diederik van de Beek, Susan J. M. Hahné, Arie van der Ende, Merijn W. Bijlsma

**Affiliations:** 1 Centre for Infectious Disease Control, National Institute for Public Health and the Environment, Bilthoven, the Netherlands; 2 Department of Neurology, Amsterdam Neuroscience, Amsterdam University Medical Centre, Amsterdam, The Netherlands; 3 Department of Medical Microbiology and the Netherlands Reference Laboratory for Bacterial Meningitis, Amsterdam UMC, University of Amsterdam, Amsterdam, the Netherlands; Centre Hospitalier Universitaire Vaudois, FRANCE

## Abstract

**Background:**

Group B *Streptococcus* (GBS) is the leading cause of neonatal sepsis and meningitis worldwide. We aimed to estimate the current burden of neonatal invasive GBS disease in the Netherlands, as a first step in providing an evidence base for policy makers on the potential benefits of a future maternal GBS vaccine.

**Methods:**

Surveillance of neonatal invasive GBS occurs at the National Reference Laboratory for Bacterial Meningitis, where culture isolates from cerebrospinal fluid and blood are sent by diagnostic laboratories. From the number of cultures we estimated the incidence of neonatal (age 0–90 days) GBS meningitis and sepsis. We constructed a disease progression model informed by literature and expert consultation to estimate the disease burden of neonatal invasive GBS infection. As many neonates with a probable GBS sepsis are never confirmed by blood culture, we further estimated the disease burden of unconfirmed cases of probable GBS sepsis in sensitivity analyses.

**Results:**

An estimated 97 cases and 6.5 deaths occurred in the Netherlands in 2017 due to culture positive neonatal invasive GBS infection. This incidence comprised 15 cases of meningitis and 42 cases of sepsis per 100.000 births, with an estimated mortality of 3.8 per 100.000 live births. A disease burden of 780 disability-adjusted life years (DALY) (95% CI 650–910) or 460 DALY per 100.000 live births was attributed to neonatal invasive GBS infection. In the sensitivity analysis including probable neonatal GBS sepsis the disease burden increased to 71 cases and 550 DALY (95% CI 460–650) per 100.000 live births.

**Conclusion:**

In conclusion, neonatal invasive GBS infection currently causes a substantial disease burden in the Netherlands. However, important evidence gaps are yet to be filled. Furthermore, cases of GBS sepsis lacking a positive blood culture may contribute considerably to this burden potentially preventable by a future GBS vaccine.

## Introduction

*Streptococcus agalactiae* or group B *Streptococcus* (GBS) is the leading cause of neonatal sepsis and meningitis. GBS is part of the microbiome of the intestinal and urogenital tract, with a carriage rate of around 20% [1]. However, after perinatal acquisition of GBS, neonates may present with signs of pneumonia, meningitis and/or sepsis. Invasive infections presenting within the first 7 days of life are categorized as ‘early-onset GBS’. Infections presenting after one week, up to three months of age, are termed ‘late-onset GBS’. Early onset disease results from vertical transmission from the colonised mother, in late onset disease the pathogen may also be transmitted by nosocomial or community sources [2].

Many industrialised countries, such as the United States, Canada and most of Europe, have adopted a GBS prevention strategy based on universal screening of pregnant women for GBS carriage, and providing intrapartum antibiotic prophylaxis when positive [3]. Other countries, including the United Kingdom and the Netherlands, do not actively screen for GBS but offer intrapartum antibiotic prophylaxis to certain risk groups, e.g. women with premature and prolonged rupture of membranes, intrapartum fever, or when GBS is cultured from urine during pregnancy [2, 4]. Antibiotic prophylaxis has important disadvantages: it exposes many women and their children to antibiotics, while only a few of the neonates would have developed invasive GBS disease [5]. Unnecessary use of antibiotics increases risks for antimicrobial resistance [6]. In addition, it can affect colonisation patterns of the neonate, which may in turn affect immunological development and body composition [7–9]. Moreover, although intrapartum antibiotics may prevent cases of early-onset GBS, it is not effective against late-onset disease [10].

A maternal vaccine against invasive GBS could overcome these disadvantages, by reducing the use of antibiotics, with likely little effect on the overall microbiome of mother and child [10]. Moreover, maternally derived vaccine-induced antibodies can remain in the child for several months, and might prevent both early and late-onset GBS disease. Several GBS vaccines are in development, but not yet on the market [11, 12]. To prepare for future introduction of GBS vaccines during pregnancy, it is important to study the current burden of neonatal GBS disease.

Recently, results were published of a comprehensive project estimating the global burden of GBS disease. This project estimated over 300.000 neonatal and infant cases of GBS disease per year worldwide, mostly occurring in low- and middle-income countries [13]. Building upon this work, supplemented by national surveillance data and studies, we aimed to estimate the burden in disability-adjusted life years (DALY) of neonatal invasive GBS infection in the Netherlands in 2017 and trends over time in 2000–2017, to serve as a baseline for future vaccine effect estimates and for prioritization of future vaccine introduction.

## Methods

### Incidence

Incidence of neonatal invasive GBS infection was estimated using data from the Netherlands Reference Laboratory for Bacterial Meningitis (NRLBM). The NRLBM receives isolates of cerebrospinal fluid (CSF) culture-confirmed cases of bacterial meningitis in the Netherlands, and collects isolates from blood cultures of patients under 1 year of age. Yearly counts of GBS isolates from patients age 0–90 days were obtained from the NRLBM register for 2000–2017. Cases were classified as sepsis (GBS cultured from blood only) or meningitis (GBS cultured from CSF only or from both CSF and blood). To estimate the coverage of this surveillance system, we used data from a previous study on neonatal GBS disease in the Netherlands, which compared the NRLBM data to a temporary enhanced surveillance system among all paediatricians nationwide in the period 1997–2001 [14]. In this study, 96 cases of proven GBS meningitis were reported by the paediatric surveillance system, and 86 were sent in to the NRLBM. As the number of received *E*. *coli* isolates from neonatal blood or CSF cultures has not changed considerably since that period, we assumed the NRLBM coverage has remained stable [2]. Therefore we used a multiplication factor of 1.12 (95% CI 1.06–1.22) to correct for the underestimation of the NRLBM data. The total number of live births per year in the period 2000–2017 was retrieved from the Statistics Netherlands open data website [15].

### Model structure

**Fig 1:**
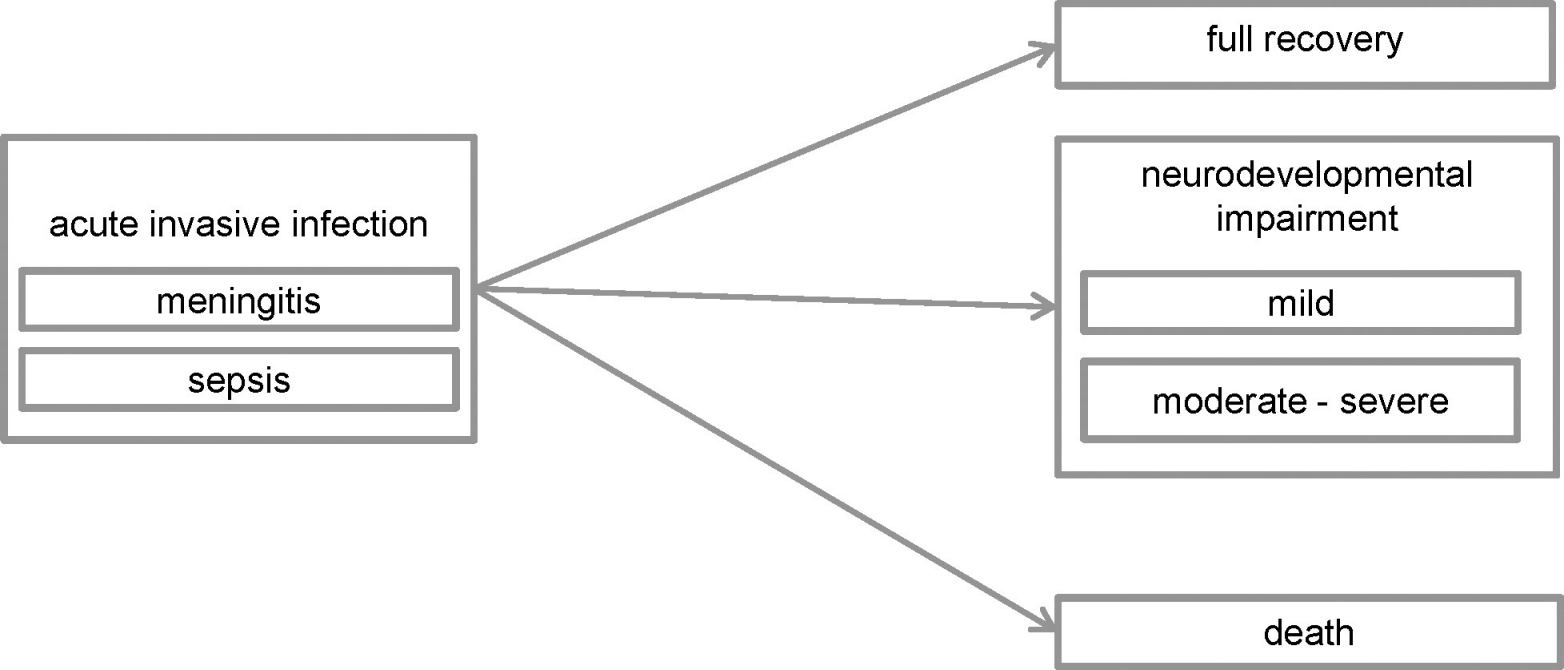
Structure of the neonatal invasive group B *Streptococcus* disease burden model.

We followed the European Centre for Disease Prevention and Control (ECDC) Burden of Communicable Disease in Europe (BCoDE) methodology, and constructed an incidence-based disease progression model [16]. As a template, the BCoDE ‘healthcare-associated neonatal sepsis’ model was used [17]. We simplified the ‘outcome tree’ by setting transition parameters to zero for unused health outcomes. Fig 1 shows the outcome tree of our neonatal invasive GBS disease model. Model structure and parameters were adjusted to the Dutch situation, informed by published and unpublished literature and expert consultations. Two practicing paediatricians with specific expertise on bacterial meningitis (authors MWB and EAMS) were consulted on the available (un)published evidence and clinical reality of neonatal GBS disease in the Netherlands. Imprecision of parameters (multiplication factors for underestimation and disease progression probabilities) was taken into account by modelling them as distributions rather than point estimates. We mainly used Beta distributions for the model parameters, however for clarification we here present the 95% confidence intervals of those distributions (Beta parameters are presented in S1 File).

### Model parameters

#### Acute invasive infection

The duration of the acute phase was derived from Schroeder et al, with 3.8 days in intensive care (ICU) and a further hospital stay of 14.7 days [18] (Table 1).

**Table 1:** Duration and disability weights for health states.

Health state	Duration	Disability weight	Sources
**Acute: ICU admission**	3.8 days	0.644 (ICU admission)	[18, 19]
**Acute: further hospital stay**	14.7 days	0.125 (infectious disease episode, severe)	[18, 19]
**Neurodevelopmental impairment: mild**	Remaining life expectancy	0.044 (motor + cognitive impairments, mild)	[19]
**Neurodevelopmental impairment: moderate—severe**	Remaining life expectancy	Uniform(0.185–0.494) (motor + cognitive impairments, moderate—severe)	[19]

#### Case fatality

We applied case fatality rates from a recently performed retrospective clinical study of all neonatal GBS cases reported to the NRLBM in a 22-year period. This unpublished study by Van Kassel *et al* found a 3-week mortality rate of 8.3% for meningitis and 6.1% for sepsis (personal communication, authors MNvK and MWB) (Table 2). These findings are in line with the estimated European GBS mortality rate of 7% [20] and with the global case fatality rate of 8.4% [13].

**Table 2:** Transition probabilities.

	After meningitis	After sepsis	sources
**Neurodevelopmental impairment: mild**	14% (95% CI 12–18%)	6.6% (95% CI 2.7–15.9%)	[21]
**Neurodevelopmental impairment: moderate—severe**	18% (95% CI 13–22%)	1.6% (95% CI 0.4–8.6%)	[21]
**Death**	8.3% (95% CI 5.6–11.5%)	6.1% (95% CI 4.4–8.1%)	Van Kassel et al unpublished

#### Long-term neurodevelopmental impairment

Within the global GBS burden project, Kohli-Lynch *et al* performed a meta-analysis of neurodevelopmental impairment (NDI) after neonatal GBS disease. Of the neonatal GBS meningitis survivors, 32% (95% CI 25%–38%) showed NDI at 18 months of follow-up, including 18% (95% CI, 13%–22%) with moderate to severe NDI. This study also included preliminary data from an unpublished study by Heath *et al*, of GBS sepsis survivors. Of the neonatal GBS sepsis survivors, 5 out of 61 had any NDI and 1 of them had moderate to severe NDI. In absence of published estimates, we based the probability of NDI after both proven and probable GBS sepsis on these numbers from Heath *et al* (Table 1).

For estimation of the years lived with disability (YLD), disability weights by Haagsma *et al* were applied [19]. The standard GBD2010 life expectancy table was used to estimate years of life lost (YLL) [22]. The models were run for the year 2017 with 10,000 iterations in BCoDE toolkit version 1.4, without discounting (except for sensitivity analysis, see below) [23]. Summation of DALY over the different models (GBS meningitis and sepsis) was done in R statistical software by simulating random draws with the assumption of normal DALY distributions. Model results were rounded to two significant digits to avoid false precision.

Model estimates of the YLD and YLL per case, estimated incidence per year from the NRLBM surveillance data and numbers of live births per year were used to estimate the DALY per 100.000 live births attributable to neonatal invasive GBS infection for the period 2000–2016.

### Sensitivity analysis

The number of isolates cultured from blood or CSF will be an underestimation of the total incidence of neonatal invasive GBS, as not all GBS infections will be culture-confirmed. A previous Dutch study found that including probable GBS sepsis, defined as a neonate with clinical and laboratory signs of sepsis and with GBS isolated from various sites but not from blood or CSF, would more than double the incidence of neonatal GBS sepsis [14, 24]. Indeed, in many cases of neonates with clinical and laboratory signs of sepsis, blood cultures remain negative. This can be due to several reasons; first, blood volumes from venepuncture in neonates may be insufficient for a positive blood culture. Second, blood culture may lack sensitivity for GBS: in a recent study, of 24 GBS PCR-positive blood samples only 13 had a positive blood culture [25]. Furthermore, it is recognised that intrapartum antibiotic treatment may lead to negative cultures despite invasive disease [24, 25]. However, culture is still the golden standard for diagnosis of invasive GBS infection, and PCR testing is not routinely performed nor are PCR results systematically collected. For these reasons, incidence and disease burden estimates for *probable* GBS sepsis (i.e. unconfirmed by blood culture but with clinical and laboratory signs of sepsis and GBS cultured from normally non-sterile sites) were included as sensitivity analysis. In the first sensitivity analysis, all probable GBS sepsis cases were assumed to be true cases of GBS sepsis. The number of received blood cultures was multiplied by 2.19 (95% CI 2.04–2.35); derived from 430 proven GBS sepsis cases of a total of 942 (proven + probable) GBS sepsis cases in Trijbels-Smeulders *et al* 2007 [14]. In the second sensitivity analysis, we assumed that a proportion of the probable GBS sepsis cases are true cases of GBS sepsis. To estimate this proportion, the case fatality rates of proven and probable GBS sepsis from Trijbels-Smeulders *et al* were compared. Because the case fatality rate of probable GBS cases was 26% (95% CI 12–51%) of the case fatality of the proven sepsis cases, we assumed 26% of probable GBS sepsis cases to be true GBS sepsis cases. Lastly, a sensitivity analysis was conducted with 3% per year discounting of DALY, with incidence based only on proven sepsis and meningitis.

## Results

### Incidence and disease burden, 2017

An overview of model results is presented in Table 3. The incidence of neonatal invasive GBS infection in the Netherlands in 2017 was estimated at 57 per 100.000 live births, or 97 cases in total in 2017. The estimated mortality of neonatal invasive GBS infection was 6.5 in total in 2017, with 1.2 deaths per 100.000 live births for GBS meningitis and 2.6 deaths per 100.000 live births for GBS sepsis. A total of 780 DALY (95% CI 650–910) was attributed to neonatal invasive GBS infection in the Netherlands in 2017; 550 years of life lost due to neonatal mortality (YLL) and 230 years lived with disability (YLD).

**Table 3:** Model results for the disease burden of neonatal invasive GBS disease burden in the Netherlands, 2017, presented as medians with 95% confidence interval.

	number of cases	number of deaths	YLD	YLL	DALY	incidence per 100.000 live births	mortality per 100.000 live births	DALY per 100.000 live births
**Neonatal GBS meningitis**	25 (24–26)	2.1 (1.6–2.6)	140 (95–190)	180 (130–220)	320 (250–390)	15 (14–15)	1.2 (0.9–1.5)	190 (150–230)
**Neonatal GBS sepsis**	72 (69–76)	4.4 (3.5–5.4)	87 (36–173)	380 (300–470)	460 (370–580)	42 (41–45)	2.6 (2.1–3.2)	270 (220–340)
**Total neonatal invasive GBS**	97 (93–101)	6.5 (5.4–7.6)	230 (140–310)	550 (460–650)	780 (650–910)	57 (55–59)	3.8 (3.2–4.5)	460 (380–540)
**Sensitivity analysis**								
**1. Total including all probable GBS sepsis**	180 (170–190)	12 (9.6–14)	330 (170–490)	1000 (820–1200)	1300 (1100–1600)	110 (100–110)	6.9 (5.7–8.3)	790 640–940
**2.Total including 26% of probable GBS sepsis**	120 (110–130)	8.0 (6.5–9.5)	260 (150–360)	680 (560–800)	940 (770–1100)	71 (66–77)	4.7 (3.8–5.6)	550 (460–650)
**3.Total (meningitis plus sepsis)– 3% discounting**	97 (93–101)	6.5 (5.4–7.6)	85 (53–120)	200 (170–240)	290 (240–340)	57 (55–59)	3.8 (3.2–4.5)	170 (140–200)

GBS: Group B Streptococcus; DALY: disability-adjusted life year; YLL: years of life lost; YLD: years lived with disability

### Trends over time, 2000–2017

**Fig 2:**
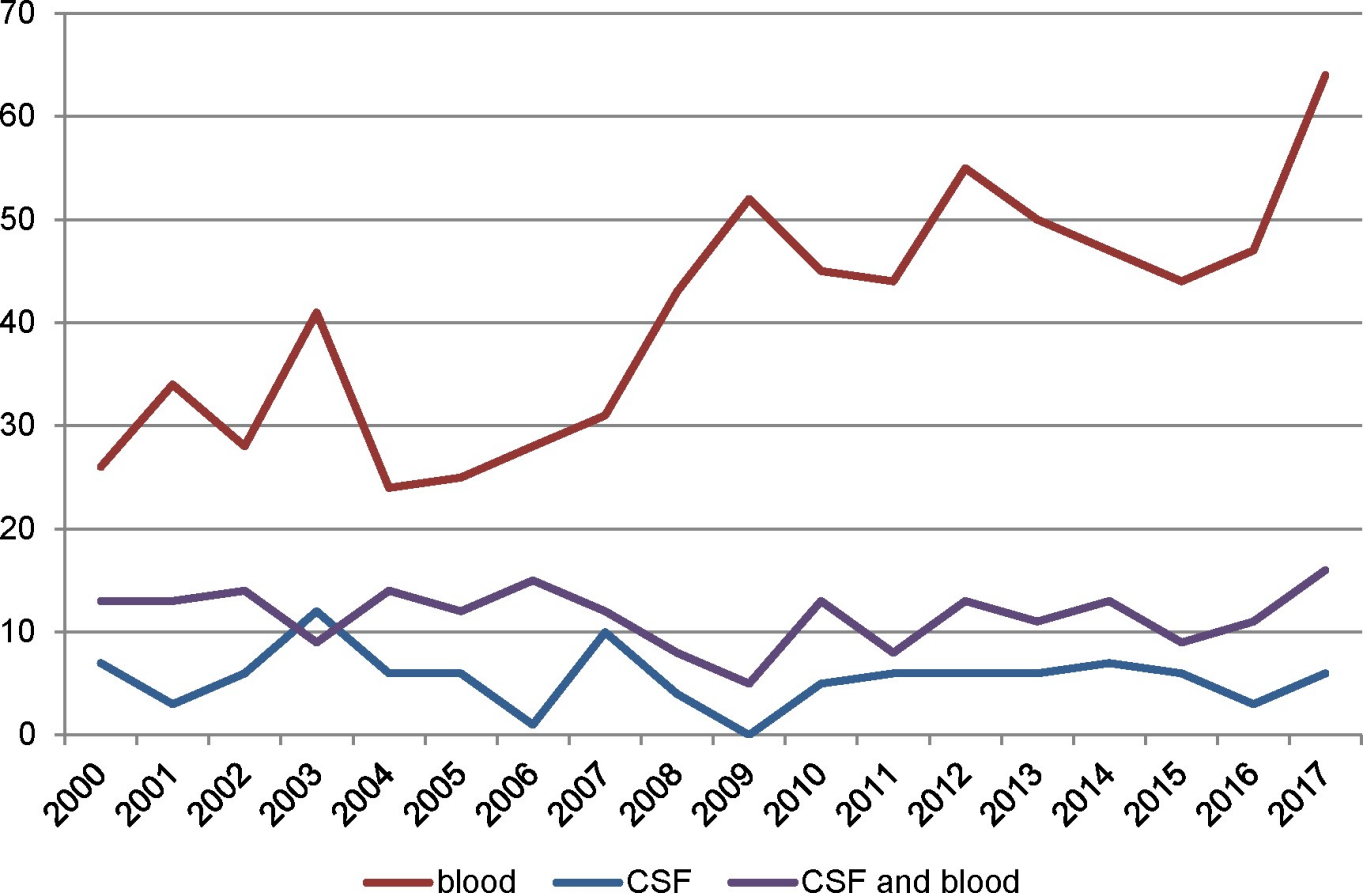
Number of GBS isolates from neonates (age 0–90 days) received by the NRLBM, by culture specimen type. CSF: cerebrospinal fluid; GBS: group B Streptococcus; NRLBM: Netherlands Reference laboratory for Bacterial Meningitis.

**Fig 3:**
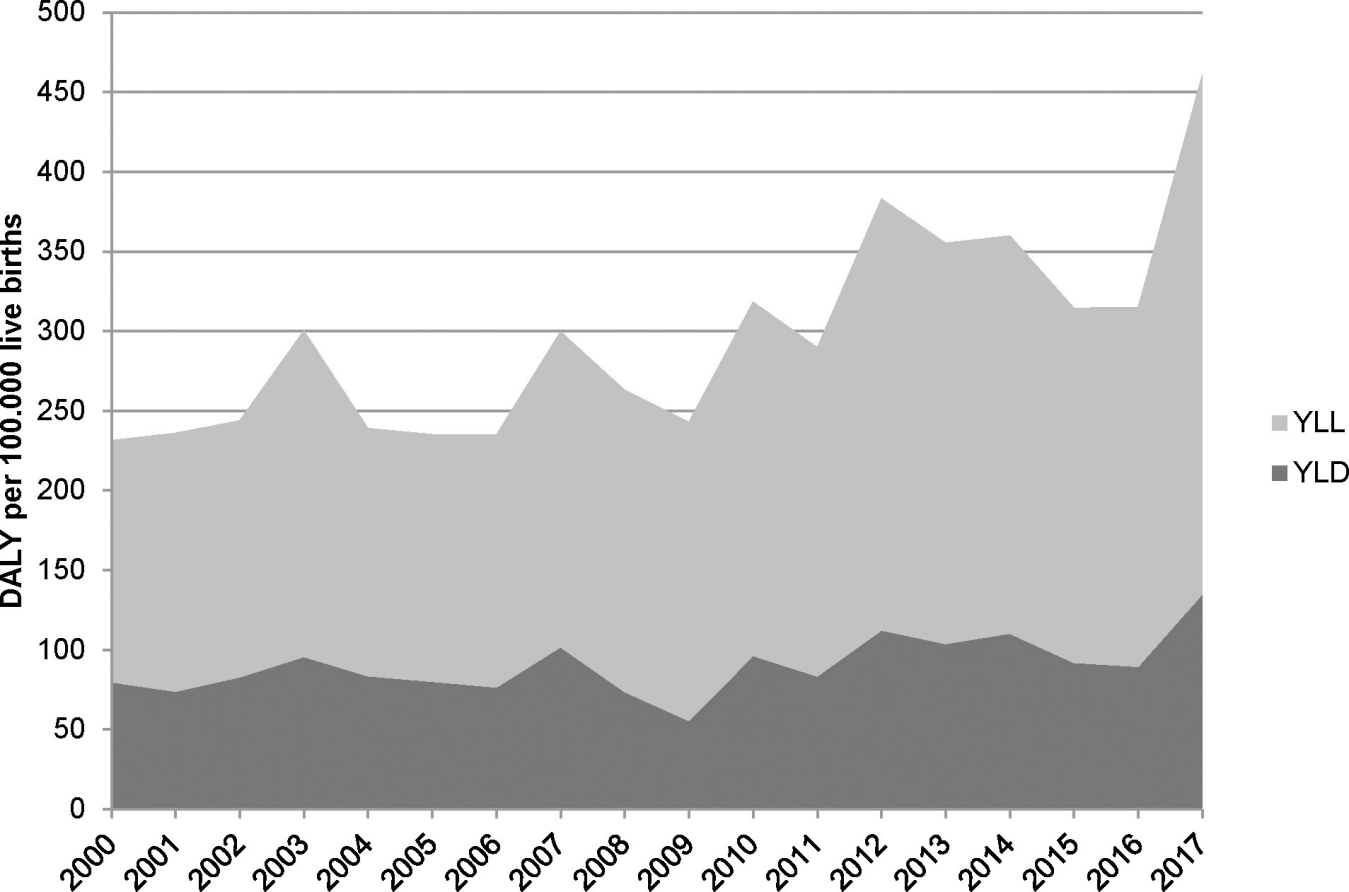
Estimated disease burden of neonatal invasive GBS infection per 100.000 live births in the Netherlands, 2000–2017, undiscounted. YLL: years of life lost; YLD: years lived with disability; DALY: disability-adjusted life year.

Fig 2 shows that the number of GBS isolates received by the NRLBM from children ≤90 days old increased over time, particularly for blood cultures. Fig 3 shows the DALY per 100.000 live births attributable to neonatal invasive GBS infection in the period 2000–2017. An increasing trend is visible, which is mostly an increase in YLL as fewer YLD are attributed to sepsis cases (estimated from blood cultures) than to meningitis (estimated from CSF or both CSF and blood cultures).

### Sensitivity analysis

**Fig 4:**
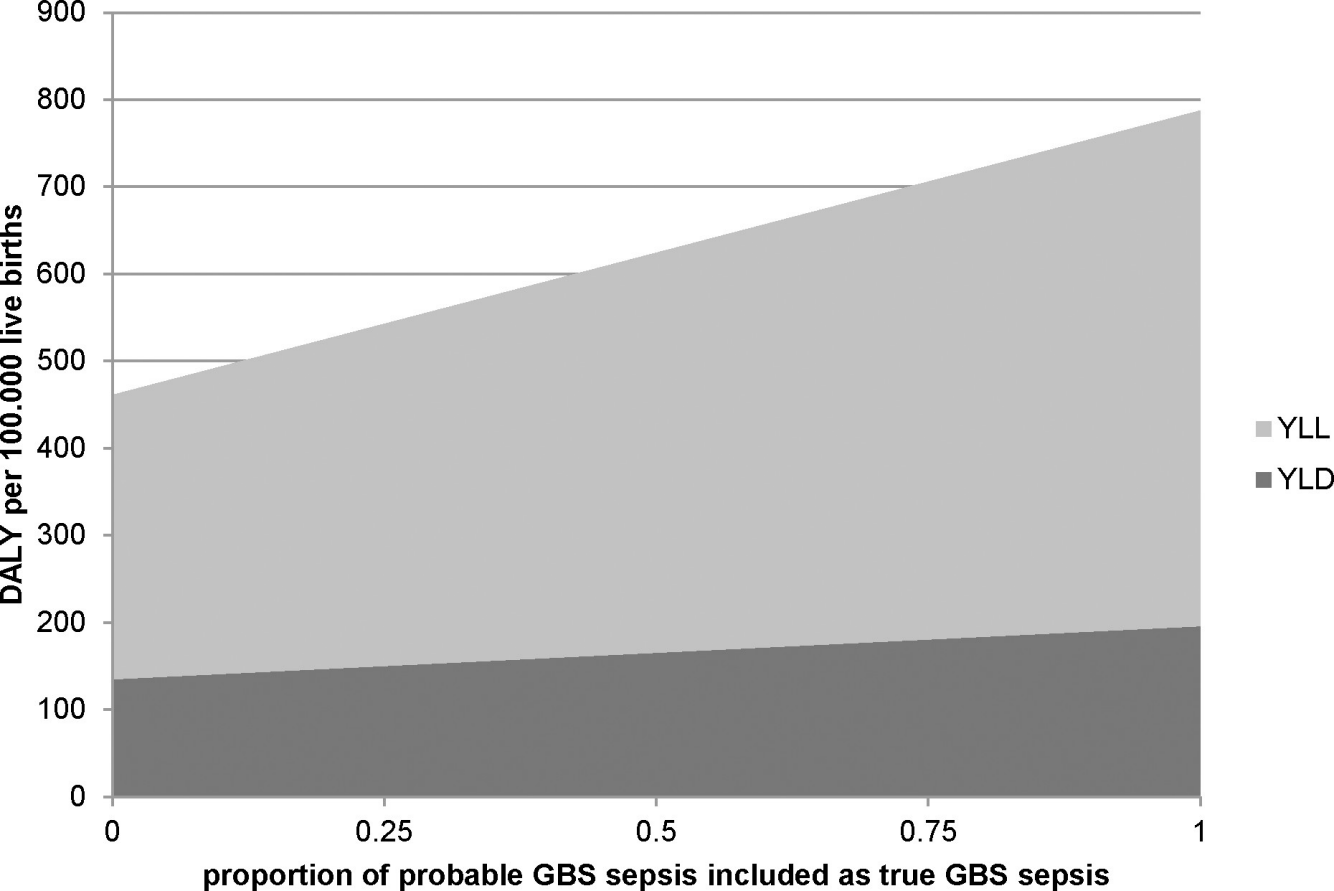
Estimated disease burden of neonatal invasive GBS infection per 100.000 live births in the Netherlands, 2017, per proportion of probable GBS sepsis cases classified as true GBS sepsis. YLL: years of life lost; YLD: years lived with disability; DALY: disability-adjusted life year; GBS: Group B *Streptococcus*.

Fig 4 shows the total neonatal invasive GBS infection YLL and YLD per 100.000 live births as function of the included proportion of neonatal sepsis cases without blood culture confirmation but with GBS isolated from normally nonsterile sites (probable GBS sepsis). When all probable GBS sepsis cases are assumed to present true GBS sepsis cases, the disease burden increases to 1300 DALY for the Netherlands in 2017 (Table 3). When a proportion of 26% of the probable GBS sepsis cases is assumed to be attributable to GBS, the total neonatal invasive GBS burden amounts to 940 DALY. When a 3% per year time discount rate is applied, 290 (95% CI 240–340) DALY are estimated for proven neonatal invasive GBS disease in the Netherlands in 2017 (Table 3).

## Discussion

We present the first estimate of the disease burden of neonatal invasive GBS infection in the Netherlands, at 780 DALY (95% CI 650–910) for the year 2017, or 460 DALY (95% CI 380–540) per 100.000 live births. This estimate is based on an estimated incidence of neonatal invasive GBS infection of 57 per 100.000 live births (95% CI 55–59), with an estimated mortality of 3.8 per 100.000 live births (95% CI 3.2–4.5). When a proportion (26%) of neonatal sepsis cases unconfirmed by blood culture but with proven GBS colonisation is included in the estimate, 940 DALY (95% CI 770–1100) or 550 DALY (95% CI 460–650) per 100.000 live births are attributable to neonatal invasive GBS infection. Surveillance data show an increasing trend of neonatal invasive GBS infections, as reported before in the Netherlands and the UK [2, 26]. The current risk factor-based GBS prevention strategy appears inadequate to halt the increase of neonatal GBS sepsis.

Our results allow for comparison of the neonatal invasive GBS disease burden with other potentially vaccine-preventable diseases in the Netherlands before introduction of a vaccine. Van Lier *et al* recently estimated the burden in DALY of several diseases in the year before introduction into the Dutch National Immunisation Programme (NIP), plus the 2017 disease burden for several candidate NIP target diseases [27]. The neonatal invasive GBS disease burden of 780 DALY is comparable to the 2017 population burden of meningococcal B (620 DALY, not included in NIP) and W disease (410 DALY, vaccine introduced in NIP in 2018), and measles (560 DALY) before the vaccine was introduced in the NIP in 1976 (S1 Fig).

Our burden estimate was restricted to neonatal invasive GBS disease only. The recent global GBS burden study included maternal disease and stillbirth [13]. We do not have national data to estimate GBS disease burden in pregnancy or the puerperium, or its impact on pregnancy outcome. Applying the incidence of maternal GBS sepsis in developed countries of 0.23 per 1000 live births, an estimated 39 cases of maternal GBS sepsis would have occurred in the Netherlands in 2017 [28]. Further, neonatal GBS pneumonia likely causes an additional substantial disease burden in the Netherlands. As cultures from sites other than blood and CSF are not routinely collected by the NRLBM, we did not have data to include pneumonia, or rare presentations such as soft tissue infections, in our estimates. A study from the UK, with a comparable incidence and ratio of GBS sepsis to meningitis, reported a further 20% of neonatal GBS disease presenting as pneumonia [29]. In addition, GBS can cause invasive disease in non-pregnant adults, which adds on to the GBS burden in the Netherlands [30].

There are some important evidence gaps on the neonatal GBS disease burden. The risk estimates of NDI after neonatal GBS disease are uncertain. NDI is a very heterogeneous outcome, which can manifest as e.g. poor academic outcomes or severe visual or hearing impairment. In a cohort of Dutch childhood meningitis survivors, 39 / 93 (42%) had academic or behavioural limitations [31]. Although this cohort included only 2–3% GBS meningitis and the age at infection was generally quite high (2–3 yo), these outcomes seem generally in line with the 32% estimated by Kohli-Lynch *et al* in the global GBS burden project [21]. However, the risk of NDI after sepsis without meningitis is more uncertain. From Kohli-Lynch *et al*., reporting preliminary results from Heath *et al*, we applied the 6.6% risk of mild NDI after GBS sepsis, plus 1.6% moderate to severe NDI. However, Haller *et al* estimated the risk of a low mental development index after neonatal sepsis in very low birth weight infants at 13%, cerebral palsy at 8%, visual impairment at 9% and hearing impairment at 4% [32]. More data is needed to quantify the risk of NDI attributable to neonatal GBS sepsis. Further, we applied the GBS meningitis model with its high NDI risk to CSF culture-positive neonates only. However, in clinical practice a lumbar puncture is not routinely performed in early onset disease. As a consequence, we will have misclassified a number of early-onset meningitis patients as sepsis patients or missed the infection altogether.

Our disease burden estimate focuses only on illness and outcomes for the child itself. However, each case of neonatal GBS disease can be expected to pose a substantial burden on families as well. Even when a case fully recovers, parents experience substantial stress and anxiety during NICU admission of their newborn [33]. In the longer term, grief after the loss of a child or worries about child development can also be expected to cause substantial distress.

In conclusion, neonatal invasive GBS infection poses a substantial disease burden in the Netherlands. The neonatal GBS disease burden is likely to be underestimated when including only cases with GBS cultured from normally sterile sites. More research is needed to quantify risk of long-term sequelae after neonatal GBS sepsis. Especially with the prospect of a maternal vaccine becoming available in the near future, a solid evidence base is needed to inform public health policy on GBS prevention.

## Supporting information

S1 Fig Ranking of vaccine-preventable diseases by estimated disease burden (expressed in DALYs) at population and individual level. Adapted from: Van Lier A, De Gier B, McDonald SA, Mangen MJJ, Van Wijhe M, Sanders EAM, Kretzschmar ME, Van Vliet H, De Melker HE. Disease burden of varicella versus other vaccine-preventable diseases before introduction of vaccination into the National Immunisation Programme in the Netherlands. Eurosurveillance. 2019; 24(18).
(PDF)

S1 File Model parameters. (XLSX)

S2 File Neonatal GBS sepsis model import file for BCoDE toolkit version 1.4. (XLS)

S3 File Neonatal GBS meningitis model import file for BCoDE toolkit version 1.4. (XLS)
