# Supplementary material for: Disease burden of neonatal invasive Group B Streptococcus infection in the Netherlands
Source: PLoS One. 2019 May 9;14(5):e0216749. doi: 10.1371/journal.pone.0216749 (PMC6508726; doi:10.1371/journal.pone.0216749)
Supplement: S1 Fig — Adapted from: Van Lier A, De Gier B, McDonald SA, Mangen MJJ, Van Wijhe M, Sanders EAM, Kretzschmar ME, Van Vliet H, De Melker HE. Disease burden of varicella versus other vaccine-preventable diseases before introduction of vaccination into the National Immunisation Programme in the Netherlands. Eurosurveillance. 2019; 24(18). (PDF) [file pone.0216749.s001.pdf]

S1 Fig. Ranking of potentially vaccine-preventable diseases by estimated disease burden (expressed in DALYs) at population and individual level. Adapted from: Van Lier A, De Gier B, McDonald SA, Mangen MJJ, Van Wijhe M, Sanders EAM, Kretzschmar ME, Van Vliet H, De Melker HE. Disease burden of varicella versus other vaccine- preventable diseases before introduction of vaccination into the National Immunisation Programme in the Netherlands. Eurosurveillance. 2019; 24(18).

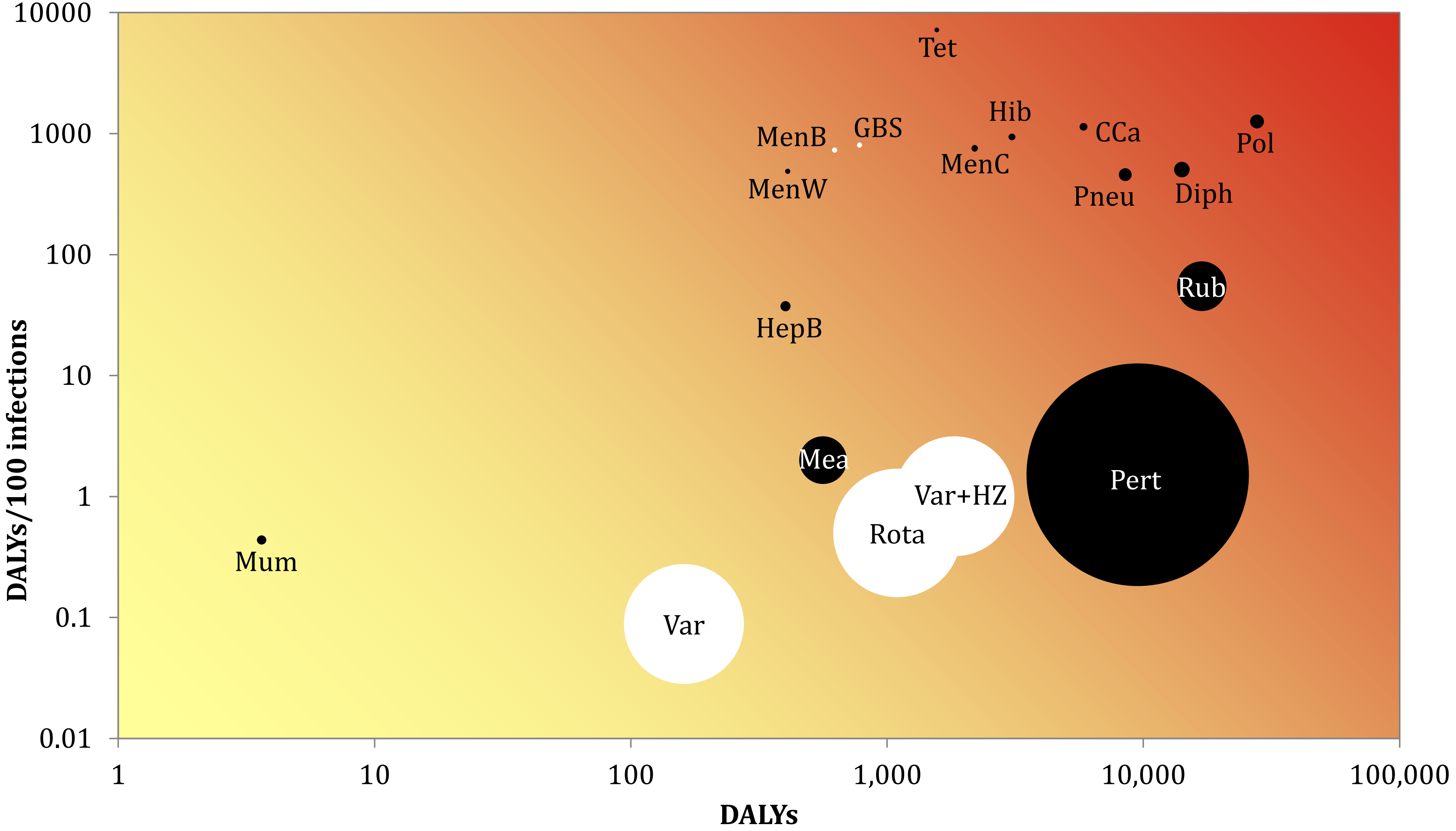

The area of each bubble is proportional to the average number of estimated cases (250 cases were added to each bubble for visibility reasons). Both axes are on a logarithmic scale.

DALY = Disability-Adjusted Life Years; black bubbles: estimates for the year before inclusion in the National Immunisation Programme (NIP), white bubbles: estimates for 2017 for potential NIP candidates.

Diph=diphtheria, Pert=pertussis, Tet=tetanus, Pol=poliomyelitis, Rub=rubella, Mea=measles, Mum=mumps, Hib=invasive Haemophilus influenzae type b disease, MenC/W/B=invasive meningococcal C/W/B disease, Pneu=invasive pneumococcal disease (PCV10 types), CCa=cervical cancer (HPV-16/18), HepB=hepatitis B, Rota=rotavirus gastroenteritis, Var=varicella, HZ=herpes zoster, GBS= neonatal invasive group B streptococcal disease (no vaccine available yet).
